# Balancing with Vibration: A Prelude for “Drift and Act” Balance Control

**DOI:** 10.1371/journal.pone.0007427

**Published:** 2009-10-20

**Authors:** John G. Milton, Toru Ohira, Juan Luis Cabrera, Ryan M. Fraiser, Janelle B. Gyorffy, Ferrin K. Ruiz, Meredith A. Strauss, Elizabeth C. Balch, Pedro J. Marin, Jeffrey L. Alexander

**Affiliations:** 1 Joint Science Department, The Claremont Colleges, Claremont, California, United States of America; 2 Sony Computer Science Lab, Tokyo, Japan; 3 Stochastic Dynamics Laboratory, Center for Physics, IVIC, Caracas, Venezuela; 4 Laboratory of Physiology, European University Miguel de Cervantes, Valladolid, Spain; 5 Department of Interdisciplinary Health Sciences, A. T. Still University, Mesa, Arizona, United States of America; Mount Sinai School of Medicine, United States of America

## Abstract

Stick balancing at the fingertip is a powerful paradigm for the study of the control of human balance. Here we show that the mean stick balancing time is increased by about two-fold when a subject stands on a vibrating platform that produces vertical vibrations at the fingertip (0.001 m, 15–50 Hz). High speed motion capture measurements in three dimensions demonstrate that vibration does not shorten the neural latency for stick balancing or change the distribution of the changes in speed made by the fingertip during stick balancing, but does decrease the amplitude of the fluctuations in the relative positions of the fingertip and the tip of the stick in the horizontal plane, A(x,y). The findings are interpreted in terms of a time-delayed “drift and act” control mechanism in which controlling movements are made only when controlled variables exceed a threshold, i.e. the stick survival time measures the time to cross a threshold. The amplitude of the oscillations produced by this mechanism can be decreased by parametric excitation. It is shown that a plot of the logarithm of the vibration-induced increase in stick balancing skill, a measure of the mean first passage time, versus the standard deviation of the A(x,y) fluctuations, a measure of the distance to the threshold, is linear as expected for the times to cross a threshold in a stochastic dynamical system. These observations suggest that the balanced state represents a complex time–dependent state which is situated in a basin of attraction that is of the same order of size. The fact that vibration amplitude can benefit balance control raises the possibility of minimizing risk of falling through appropriate changes in the design of footwear and roughness of the walking surfaces.

## Introduction

The maintenance of balance while standing and during locomotion arises from complex interactions between the walker and their environment. So robust are the control mechanisms that the occurrence of a fall is a cause of great concern to the walker and often to the medical profession as well. Current hypotheses for the control of balance are motivated by considerations of the stabilization of a pendulum in the inverted position; a classic problem in control theory [1]. The control problem arises because the upright position of the pendulum is unstable and hence even the slightest perturbation is sufficient to cause it to fall over. Consequently, for over 20 years, it has been assumed that human balance represents an equilibrium that is stabilized by the interplay between the biomechanical properties of the musculo–skeletal systems [2]–[4] and by time–delayed negative neural feedback [5], [6]. Recently this view of human balance control has been challenged [7]–[9]. A growing number of experimental observations emphasize that the upright position is not a stable equilibrium, but a more complex and bounded time–dependent state [4], [10]–[16]. Moreover, control in two paradigms of human balance control, namely stick balancing at the fingertip [7], [17] and postural sway during quiet standing [8], [9], [18], [19], is intermittent not continuous.

A direct way to explore the nature of the balanced state is to examine the effects of parametric excitation on the ability of a subject to balance a stick at their fingertip. The term ‘parametric excitation’ refers to the fact that when the pivot point of an inverted pendulum is vibrated the effects enter the equations of motion through a time varying parameter [20], [21]. It is well known that if the upright position is an equilibrium then it can be stabilized by vibrating the pivot point in the vertical direction using frequencies that exceed
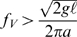
(1)where 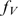 is the critical frequency (cycles/sec) at which the upright position is stabilized, 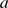 is the peak–to–peak amplitude of the oscillation, 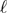 is the length of the pendulum, and 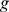 is the acceleration due to gravity [20]. However, this effect requires that the pivot point be physically attached to the pendulum in order that downward accelerations exceed gravity [21]: this is not possible for stick balancing since the stick sits on the fingertip but is not bonded to it. An alternate hypothesis for balance control, referred to herein as “drift and act”, is that the desired upright position is one in which the dynamics are confined within a small basin of attraction: inside the basin of attraction trajectories “drift”; however, whenever trajectories exceed the basin boundaries, corrective actions (“act”) are taken to redirect the trajectories back into it [7], [13], [16], [22]–[27]. The observed statistical properties of human stick balancing, namely the Weibull–type survival functions [28], [29], the 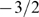 power laws that describe the times between successive corrective movements [17], and the Lévy distributions which describe the changes in speed made by the fingertip [30], [31], point to an underlying dynamical system that is tuned near enough to the edge of stability that critical control parameters can be noisily forced back and forth across the stability boundary. In the setting of drift and act control any amplitude lowering effect due to parametric excitation would be stabilizing since it biases the fluctuations away from the stability, or basin, boundary and hence prolongs the first passage time.

**Figure 1:**
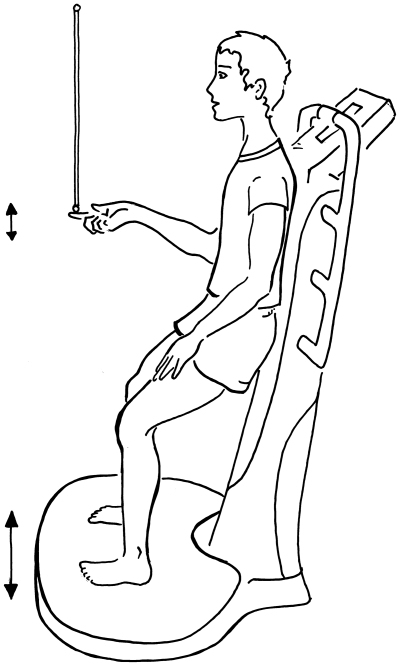
Schematic representation of stick balancing at the fingertip while standing on a vibrating platform. A schematic representation of stick balancing at the fingertip while standing on a vibrating platform. The subject used the back support of the vibrating platform to help stabilize their posture. The subjects self–selected the degree of flexion of their knee for comfort.

The organization of our discussion is as follows. First, we demonstrate that the mean stick balancing time is increased when the subject stands on a vibrating platform (Figure 1). The purpose of the vibrating platform is to introduce periodic vertical vibrations at the fingertip (parametric excitation) in a manner that does not influence the freedom of the balancing arm and hand movements. Second we show that whole body vibration does not decrease the neural latency for stick balancing or alter the changes in speed made by the fingertip during stick balancing. Third it is shown that the relative movements of the position of the fingertip and tip of the stick exhibit an oscillatory relationship in the horizontal plane and that vibration decreases the amplitude of these fluctuations. Fourth, it is shown that a plot of the logarithm of the vibration-induced increase in the mean stick balancing time, a measure of the mean first passage time, versus is the standard deviation of these fluctuations, a measure of the distance to the threshold, is linear as expected for the times to cross a threshold in a stochastic dynamical system [32], [33]. Finally, we illustrate that for a generic class of “drift and act”–type mathematical models parametric excitation can produce a lowering of the amplitude of limit cycle oscillations.

## Results

**Figure 2:**
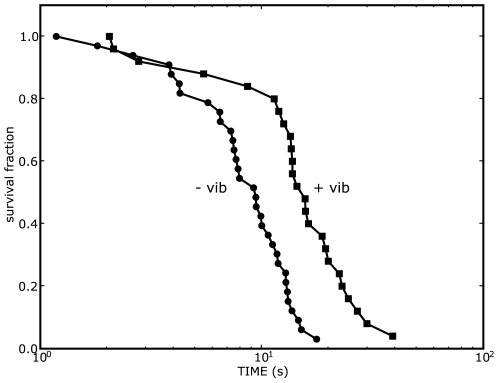
Vibration enhances stick balancing skill. The survival fraction represents the fraction of stick balancing trials for which the stick was still balanced at time 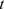 (see METHODS for more details): ‘
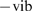
’ means no vibration and ‘
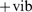
’ means with vibration. The survival fraction is determined using 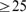 stick balancing trials and the mean survival time, 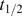, is used as a measure of stick balancing skill. Here a 50 Hz, 0.001 m peak–to–peak amplitude vibration at the fingertip approximately doubles the mean survival time (see Figure 3 and Table 1 for summary of results).

Subjects balanced a stick on their fingertip while standing on a vibrating platform (Figure 1). Figure 2 shows the effect of a 50 Hz, 0.001 m vertical vibration at the fingertip on the stick survival curve for one subject. The stick survival curves have the characteristic shape expected for a failure time process [28], [29]. The effect of the vibration is to shift the stick survival curve to the right. For the subject in Figure 1 the mean stick survival time, 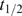, a measure of balancing skill, is increased by 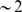
–fold (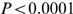, Mann-Whitney U test).

**Figure 3:**
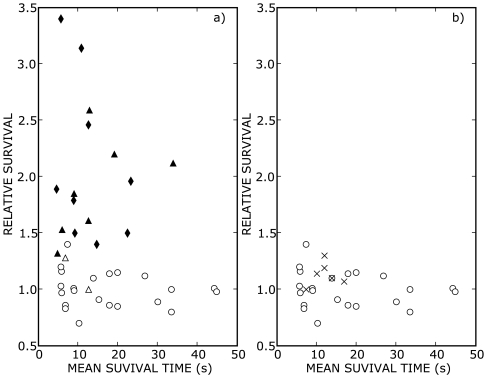
Effect of vibration amplitude and frequency on the mean stick balancing time. a) shows the effect of 0.001 m vertical vibration at the fingertip on relative survival and b) shows the effects of whole body vibration on relative survival using a vibrating platform which vibrated the body in a way that did not produce detectable vertical vibrations at the fingertip. The relative survival is the mean stick survival time, 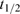, measured for stick balancing in the presence of vibration divided by that obtained in the absence of vibration. In a) the shape of the symbol indicates the vibration frequency; 15 Hz (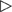), 25 Hz (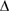) and 50 Hz (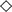), and filled symbols indicate a statistically significant enhancement in stick balancing skill (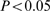). In b) the relative survival of subjects (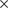) was not significantly enhanced by whole body vibration (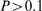 in all cases).

The effects of 15–50 Hz vibration on stick balancing skill for 9 subjects having a range of skill levels are summarized in Figure 3 and Table 1. Two types of vibrating platform were used (Table 1): vertical vibrating platforms that produce periodic vertical vibrations at the fingertip (Physioplate, iTonic, Powerplate) and a vibrating platform that produces aperiodic vibrations mainly in the horizontal plane (Soloflex). Figure 3a shows that for 19/21 experiments using the vertical–type vibrating platforms, vibration produced a statistically significant improvement in stick balancing skill (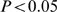; in 11/19 experiments the level of significance was 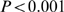). In 2/21 experiments (open 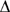) the increase in mean survival time with vibration did not reach the level of significance (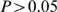). These experiments involved two subjects vibrated at 25 Hz: in each case a statistically significant increase in stick balancing skill was obtained when the vibration frequency was increased to 50 Hz.

**Table 1:** Vibration characteristics and stick balancing skill.

	Vibration Amplitude (mm)			Relative Survival
Vibration Source	Sole	Fingertip (still)	Fingertip (Balancing)	
None (18) 	-	-	-	1.0 (0.8–1.2) 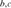
Physioplate				
15 Hz (3)	2.3	0.3	0.1	2.6 (1.4–4.1)
25 Hz (3)	1.2	0.2	0.1	2.1 (1.5–2.6)
50 Hz (3)	0.9	0.1	0.1	2.2 (1.5–3.1)
iTonic/Powerplate				
25 Hz (6)	1.1	0.1	0.1	1.5 (1.0–2.1)
50 Hz (6)	1.2	0.1	0.1	2.1 (1.4–3.4)
Soloflex (6)	0.3	0.1	UD 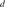	1.0 (0.9–1.2)


 Number of subjects given in brackets.

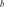
 Mean (Minimum value - Maximum value).

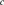
 Relative survival is the mean stick survival time measured in the presence of vibration divided by the mean stick survival time measured in the absence of vibration.

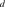
 UD is undetectable.

In contrast, Figure 3b shows that when 7 subjects performed stick balancing while standing on a vibrating platform that produced undetectable vertical vibrations at the fingertip, no statistically significant enhancement of skill was observed (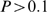 for all subjects). Taken together, these observations strongly indicate that the vibration–enhancement of stick balancing skill is not simply due the effects of whole body vibration *per se*, for example on vision [34], but are primarily related to vertical vibrations at the fingertip.

### Vibration and neural latency

Modeling studies of an inverted pendulum controlled by time–delayed negative feedback indicate that a necessary, but not sufficient, condition for stabilization is that the neural latency, 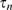, is less than a critical delay, 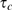, given by 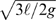 [5], [15], [35]. For 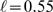 m, 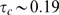 s which is longer than estimates of 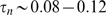 s for low to moderate skill stick balancers [30]. To test the possibility that the beneficial effects of vibration on stick balancing skill were related to its effects on neural response times, we measured the cross–correlation, 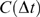, between the position of the tip of the stick at time 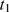 and the corrective movements made by the fingertip at time 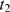, i.e. 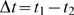 [30]. The shift in 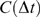 from 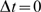 gives an estimate of the response time, or neural latency, for stick balance control.

**Figure 4:**
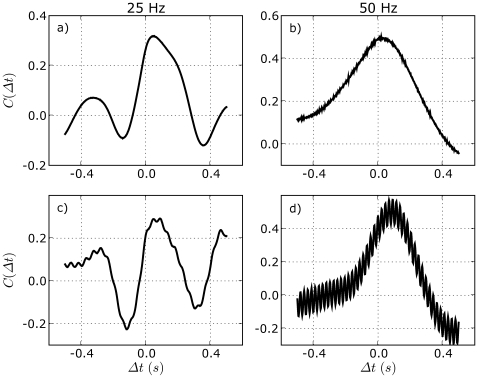
Effect of vibration on neural latency for stick balancing skill. The cross–correlation function, 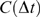 for stick balancing is measured in the absence of vibration (top panels) and in the presence of vibration (bottom panels). Data is shown for two subjects having different skill levels: in the absence of vibration 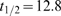 s for the subject on the left (33.2 s in presence of vibration) and 23.2 s for the subject on the right (45.5 s in presence of vibration).

Figure 4 shows the effect of vibration on 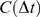 for two subjects. It can be seen that vibration shifts 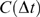 to the right by 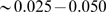 s. Thus the vibration enhancement of stick balancing skill is not due to a shorter neural latency, i.e. a faster neural response time. Increases in neural latency towards 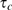 as stick balancing skill increases with practice have been observed previously [30], and have been interpreted as reflecting a decreased role for active neural control. It should be noted that since a vibratory input to the fingertip necessarily effects the position of the reflective markers at both ends of the stick equally, it cannot itself produce a shift in 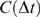. Consequently the effects of vibration are superimposed on 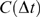. Differences between the prominence of the vibratory component to 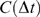 (compare Figure 4c and d) presumably reflects differences in the low–pass filtering characteristics of different bodies and postures on the vibratory input applied at the sole of the foot and were not investigated further.

### Vibration and fingertip speed

Previous studies have shown that the distribution of the changes in speed, 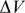, made by the fingertip during stick balancing, 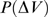 is Lévy–distributed [30], [31]. Increases in stick balancing skill over the first few days of practice are mirrored by a broadening of the tails of 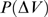. In other words, skilled stick balancers are able to make, or tolerate, larger 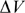
's. Whole body vibration can alter motor performance through its effects on skeletal muscle [36], muscle spindles [37]–[39] and motor cortex excitability and voluntary drive [40]. Thus it is important to determine whether the beneficial effects of vibration on stick balancing are manifested through its effects on 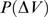.

**Figure 5:**
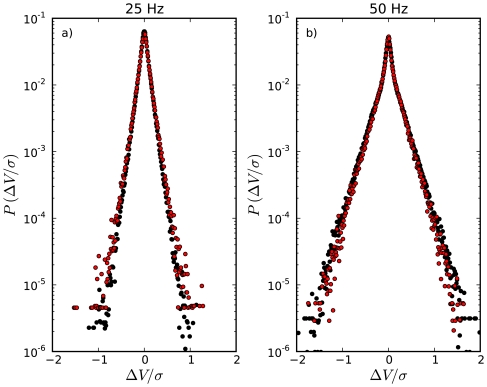
Effect of vibration on the distribution of the changes in speed made by the fingertip during stick balancing. High speed motion capture cameras were used to measure the distribution, 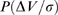, of the changes in speed, 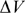, of the movements of the fingertip in the presence (red •) and absence (black •) of vibration, where 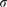 is the standard deviation. Data is shown for the same subject: the 50 Hz vibration experiment was done 2 days after the 25 Hz vibration experiment. The broadening of 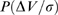 is consistent with the increase in stick balancing skill that the subject experienced: 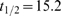 s in the absence of 25 Hz vibration and 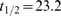 s in the absence of 50 Hz vibration. The sampling frequency was 500 Hz.

Figure 5 compares the effects of 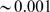 m, 25 Hz and 50 Hz vibration on 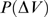 for one subject. Clearly vibration produces no significance change in 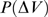 and, in particular, does not broaden the tails of the distribution. The same observations were obtained for two other subjects (one having a higher skill level and the other a lower skill level than the subject shown in Figure 5 (data not shown)). Thus the beneficial effects of vibration on stick balancing skill are not related to changes in 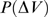.

### Vibration and fingertip movements

**Figure 6:**
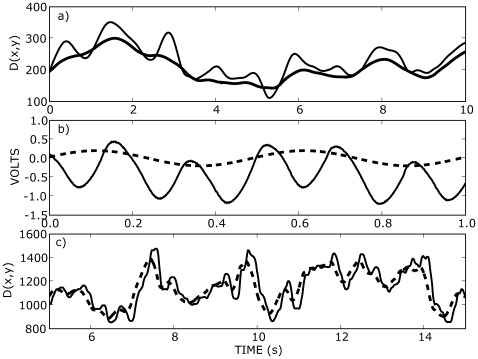
Comparison of three paradigms for stick balancing: a) stick balancing at the fingertip, b) mechanical stick balancing, and c) virtual stick balancing. In all cases the dashed lines are related to the controlled variable and the solid lines are related to the controller: a) plots the position of the fingertip tip (solid line) versus the tip of the stick (dashed line); b) plots the voltage proportional to the displacement angle (dashed line) versus the voltage response of the controller (solid line), and c) plots the position of the target (dashed line) versus the position of the computer mouse (solid line). In a) and c), 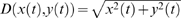 is the length of the position vector measured at time 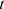 from a common reference point, 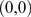, supplied, respectively, by the Qualisys motion capture system and the computer program. No ambiguity arises from the use of 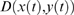 since the vertical displacement angle is small (see Figure 7d) and the movements of the fingertip and tip of the stick are necessarily strongly correlated.

An often under–appreciated aspect of stick balancing is the oscillatory relationship between the relative movements of variables related to the controlled variable, e.g. the vertical displacement angle, 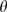, and variables related to the controller, e.g. the position of the fingertip. Experimentally this oscillatory relationship is most easily appreciated by viewing stick balancing from above looking downwards. In this view information concerning the vertical extent of the movements is necessarily lost, but the oscillatory relationship between the movements of the fingertip and the tip of the stick in their respective horizontal planes is clearly seen. We represented these movements by the calculating the length, 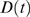, of the position vector to the fingertip or the tip of stick from a common reference point, 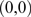, where 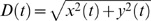. Figure 6a shows the oscillatory relationship between the movements of fingertip and tip of the stick represented in this manner (see legend for more details). This oscillatory relationship between controlled and controller is not unique to stick balancing at the fingertip but arises in mechanical stick balancing (Figure 6b), virtual stick balancing (Figure 6c), and even human postural sway [2]. The oscillatory movements are thought to be related to intrinsic difficulties in controlling both the position of the fingertip and the vertical displacement angle, 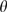, [15] and the lag arises because these paradigms in essence represent a time–delayed pursuit–escape task.

**Figure 7:**
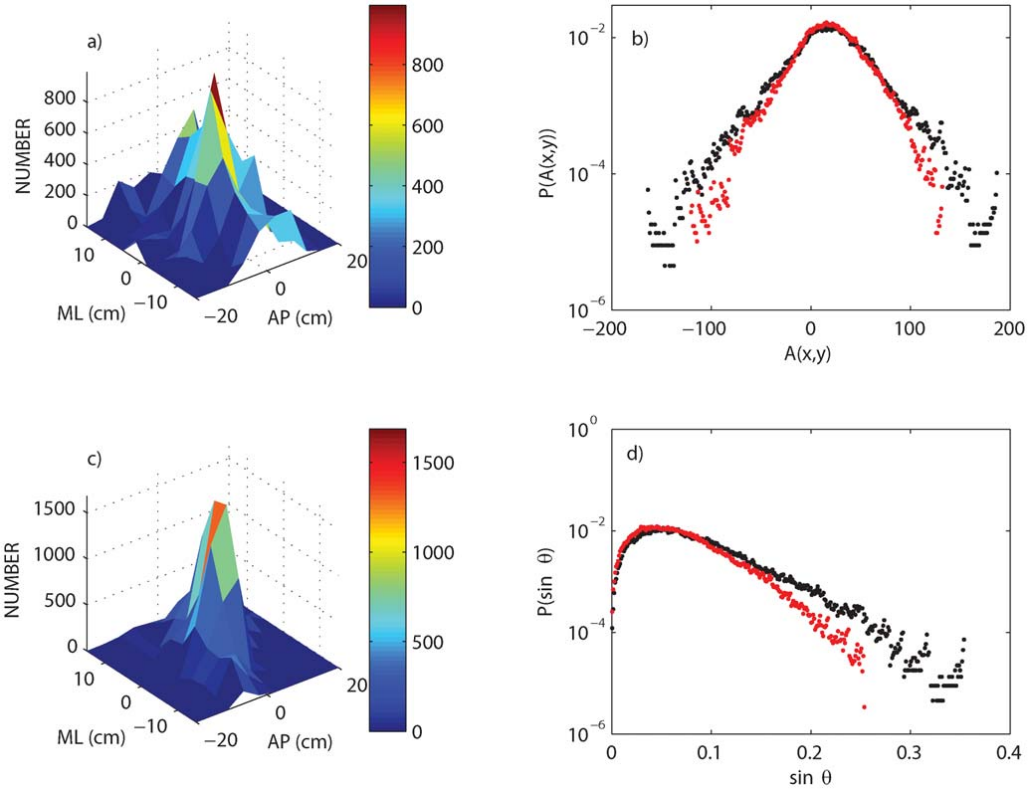
Effects of vibration on the vertical displacement angle and the amplitude of oscillatory relationship between the controlled variable and controller for stick balancing. a) and c) compare, respectively, the movements in the fingertip during stick balancing in the anterior–posterior (AP) and medial–lateral (ML) plane when the platform vibration is off and on (Physioplate vibrated at 50 Hz). These two–dimensional histograms are each determined from a single stick balancing time series of approximately equal length (39.96 s in the absence of vibration and 42.14 s in the presence of vibration). b) plots the normalized distribution of the amplitude 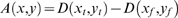 d) plots the normalized distribution of the vertical displacement angle, 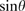 in the absence (black) and presence (red) of vibration. The subscripts 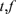 refer, respectively, to the 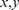 coordinates of the tip of the stick and the fingertip. The distributions shown in b) and d) are determined for a total of 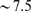 min accumulated stick balancing time in the absence of vibration and 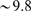 min accumulated stick balancing time in the presence of vibration (sampling frequency 500 Hz in both cases).

Figure 7 shows the effects of vibration on the movements of the fingertip and the vertical displacement angle, 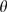, of the balanced stick. By comparing sufficiently long balancing trials of approximately the same length (see figure legend for details) it is seen that the effect of vibration is to concentrate the movements of the fingertip over a smaller area in the horizontal plane (compare Figure 7a and c). The difference between the solid and dashed lines in Figure 6a is defined as 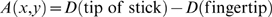. Figure 7b shows that the standard deviation of 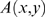 is decreased in the presence of vibration and Figure 7d shows that 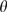 is biased towards vertical.

**Figure 8:**
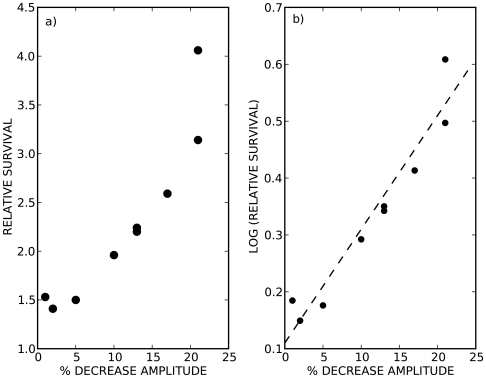
Vibration–induced enhancement of stick balancing skill as a function of vibration-induced amplitude lowering. a) and b) show the same data plotted in two different ways. Data is obtained from three subjects using three different vibration frequencies (15 Hz, 25 Hz, 50 Hz) on three different days. Relative survival is the same as defined in Figure 3. The ‘% decrease amplitude’ is calculated from the standard deviation of 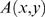 in the presence and absence of vibration, where 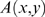 is defined in the legend to Figure 7.

Figure 8 summarizes the relationship between the vibration–induced decrease in the fluctuations in 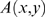 and the increase in stick balancing skill. Since the changes in 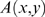 are not precisely periodic, we treated them as a stochastic signal and characterized the amplitude of the fluctuations using the standard deviation. Clearly the larger the reduction in the standard deviation of 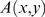, the greater the vibration–induced enhancement in stick balancing skill (Figure 8a). These observations can be re–interpreted in terms of the Kramers rate theory for the escape of a particle from a potential well [32], [33]. According to this theory the mean first passage time, 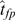, i.e. the mean time that it takes a particle to exceed the height of the potential barrier, is related to the barrier height by
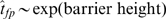
If we identify 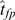 with the relative survival (RA), and the decrease in the 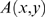 fluctuations with a vibration–induced increase in barrier height, then Figure 8b shows that a plot of the logarithm of RA versus the % decrease of the amplitude fluctuations is linear. Thus the effects of vibration of stick balancing skill can be well understood from the effects of vibration on increasing the effective barrier height of a potential well by decreasing the amplitudes of the fluctuations.

## Discussion

Our observations demonstrate that stick balancing skill can be enhanced by periodically vibrating the fingertip in the vertical direction. The frequency of these vibrations (15–50 Hz) is much less that required for the stabilization of an inverted pendulum by vibrating an attached pivot point (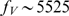 Hz for a = 0.001 m and 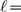 0.55 m). The whole body vibration enhancement of the mean stick balancing time is observed only when vertical vibrations are produced at the fingertip, is associated with a small increase in neural latency, and produces no changes in the distribution of the changes in speed made by the fingertip. Taken together these observations suggest that the skill enhancement is due to vertical vibrations at the fingertip and not to the effects of vibration on the nervous or musculo–skeletal system. We suggest that a simple explanation for this unexpected observation is to hypothesize that the upright balanced position is not a simple equilibrium, but represents a complex bounded time–dependent state that is confined within a basin of attraction whose size is of the same order [7]–[9], [15]. Consequently, for sufficiently large fluctuations, trajectories can escape the basin of attraction, and the stick subsequently falls. In this setting, any mechanism that biases the fluctuations generated by this time-dependent state away from the basin boundary enhances stick balancing skill. The experimentally observed exponential relationship between the vibration–induced increase in stick balancing skill and the decrease in the amplitude of the fluctuations the the fingertip-stick movements supports this interpretation.

**Figure 9:**
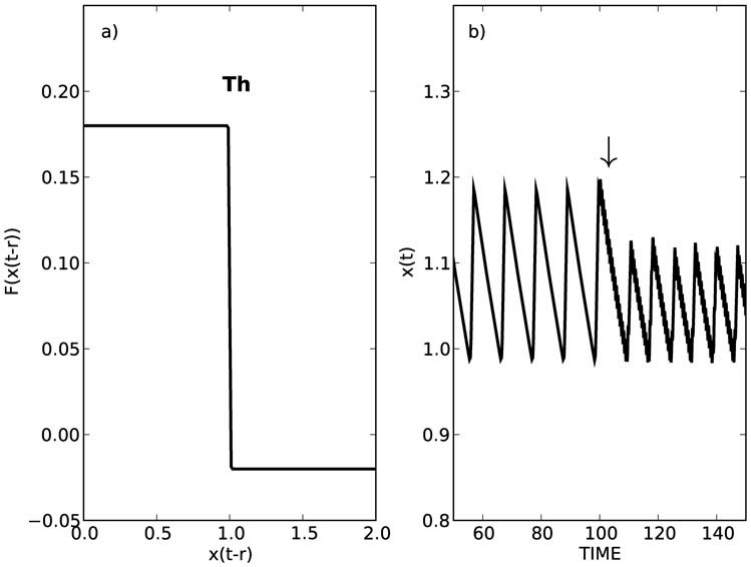
Effects of parametric excitation on the dynamics of a simple “drift and act” controller. a) Graphical representation of a simple realization of the feedback function that produces a limit cycle oscillation in (2) in the absence of parametric excitation and noisy perturbations, where 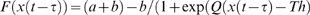 and 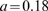, 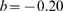, 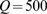, and 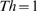. The displacement from the upright position, 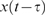, grows when 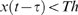 and decreases when 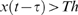. b) Periodic parametric excitation is turned on at the 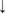. The effect is to decrease the amplitude of the limit cycle oscillation. Parameters are 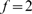 and 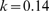.

Although, the use of parametric excitation to control the amplitude of limit cycle oscillations has been described previously [41]–[46], little attention has been previously given to the possible implications of this mechanism for human balance control. Recent control theoretic arguments for the control of an unstable fixed point in the presence of time delayed feedback and random perturbations (“noise”) have emphasized the need for switch–like controllers in which for small displacements the variable “drifts” with active control (“act”) taken only once the variable exceeds certain thresholds [7], [10], [13], [22], [23], [25]–[27]. A one–dimensional generic model with “drift–and–act” control of human balance with parametric excitation takes the form
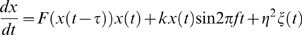
(2)where 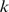 is a constant, 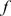 is the forcing frequency, 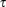 is the time delay, 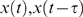 are, respectively, the values of the controlled variable at times 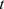 and 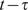, and 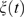 describes white additive noise with variance 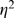. The feedback function, 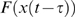, has the step–like shape shown in Figure 9a. Models of this type have been successfully employed, for example, to obtain insights into the properties of the two–point correlation functions observed for human postural sway [7], [13], [16]. Figure 9b illustrates that in the absence of noise the amplitude of a limit cycle oscillation can be lowered using parametric excitation. The attractiveness of drift and act, and related controllers, is that they are robust, inexpensive to implement, and optimal for finite corrective actions [47]. However, it may also be possible to gain further insights into our observations by examining the effects of parametric excitation on recently developed models for balancing that are based on an inverted pendulum controlled by nonlinear, time–delayed feedback [8], [9], [15], [17], [35], [48], [49].

Measurements of the frequency and amplitude dependence of the vibration–enhancement of stick balancing skill provide the direction for future model development. However, there are two intrinsic limitations of our experimental design. The first limitation arises because we use whole body vibration to introduce vibration at the fingertip. Consequently the range of frequencies available for testing is limited because frequencies less than 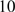 Hz are considered harmful for humans [50], [51] and those greater than 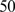 Hz were reported by our participants to be extremely uncomfortable. Similarly the range of amplitudes generated at the fingertip by vibrating the feet is limited by the fact that for vibrating frequencies above 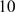 Hz the human body with knees flexed behaves as a powerful low–pass filter [50], [51]. In order to overcome these limitations it will be necessary to develop experimental techniques suitable for introducing vibrations directly to the fingertip without hindering the movements of the balancing arm and hand; possibly techniques that involve appropriately designed puffs of air.

The second, and perhaps more important, limitation is related to the assessment of stick balancing skill. Using the mean stick survival time, 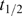, determined from at least 25 stick balancing trials as a measure of skill level corresponds to 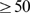 trials per vibration condition (see METHODS) and translates into 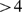 minutes accumulated exposure to whole body vibration for a subject with 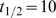 s. Thus in order to minimize fatigue, each experiment was done on a different day. However, since stick balancing is a voluntary goal–directed task, skill level increases each day with practice [52]. These changes in skill level are not detectable over the time it takes to determine 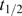, but are readily apparent when 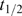 is compared from one day to the next. For example, we observed that 6 out of 11 subjects who practice stick balancing 30–60 minutes a day achieve stick balancing times 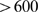 s within 14 days of consecutive practice. In view of these considerations our experiments focused on subjects who had relatively low stick balancing skill levels (e.g. 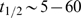 s). The observations in Figures 7a and c suggest that it might eventually be possible to assess skill from measurements made on a single, sufficiently long stick balancing trial (see legend).

It is tempting to speculate that drift–and–act control strategies might also be involved in the maintenance of postural balance. Since falls for adult humans are rare events compared to stick falls during stick balancing, the basin of attraction for posture is larger that that for stick balancing. Experimental evidence in favor of drift–and–act type human postural balance control include the intermittent nature of the corrective movements [17]–[19], the persistence and anti–persistence properties of the two–point correlation functions for postural sway [10], [13], [15], [16], and the ankle–hip–step strategies used by humans to maintain balance in response to increasingly large perturbations [53]. Indeed we have confirmed that vibration applied to bilateral Achilles' tendons during quiet standing produces a reduction in the amplitude of the fluctuations in the center of pressure during postural sway as predicted by (2) (unpublished observations).

Human movements and balance control take place in a randomly fluctuating environment. The anticipation that random fluctuations can improve balance control has already been verified [54]–[57]. Here we have shown that introducing vibrations to the body has functional benefit, namely the vibrations enhance stick balancing skill. Our observation that the amplitude of the vibrations is important for stabilizing balance raises the possibility that falls are not always simply related to “slips and trips”, but may be encouraged by modern day society's efforts to filter out effects of surface–induced vibrations through shoe and walking surface design. Thus, in view of the impending epidemic of falling due to aging demographics [58], it may be possible that changes in walking shoe and surface design may help reduce the risk of falling in this population.

## Materials and Methods

### Subjects

Data was collected and analyzed for 11 females and 7 males ages 18–59 years who were free of balance disorders. This study was approved by the institutional review board at Claremont McKenna College and A. T. Still University in accordance with the currently applicable U. S. Public Health Service Guidelines. All participants provided written informed consent for all research testing.

### Stick balancing at the fingertip

Stick balancing was performed while the subject stood on a vibratory platform in stocking feet with knees slightly flexed and their back against a vertical support (Figure 1). Sticks were wooden dowels with diameter 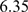 mm and length 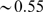
m. For each stick balancing trial we used a coin flip we used a coin flip to determine whether the vibration was on or off. This procedure was continued until we had accumulated at least 25 trials for each condition, a process that took 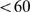 min to complete depending on the skill of subject. The time that the stick remained balanced at the fingertip was timed using a stop watch. Control studies (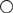 in Figure 3) mimicked this procedure except that the subject was not subjected to whole body vibration and the coin flip was used to randomly assign each trial to one of two groups.

**Stick balancing skill** was measured by estimating quantities related to the first passage time, i.e. the time elapsed until the balanced stick falls [28], [29]. The survival function, 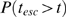, for stick balancing has the form of a Weibull survival function, i.e. 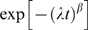, where 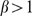. The mean stick balancing time, 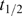, calculated using a minimum of 25 consecutive trials, was used as a measure of skill level. Participants for this study were selected from a group of subjects who had practiced stick balancing for a few days. We selected those subjects who had achieved a low to moderate skill level (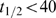
s; Figure 3). Approximately 50% of subjects achieve much higher skills levels within 10 days of practice, e.g. 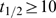 min) and were excluded since the time to complete the required 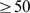 trials would have been so long (e.g. at least 8 hours) that fatigue would have become a factor.

**Vibrating platforms** were commercially available: Physioplate (Globus Sport and Health technologies, LLC), iTonic (Freemotion Fitness), Powerplate (Powerplate North America, Northbrook, Illinois) and Soloflex (Soloflex, Inc.). The frequency and vertical amplitude of the vibrations were measured at the platform surface and at the fingertip using a three camera motion capture system (Qualisys Oqus 300, sampling frequency 500 Hz). Reflective markers were firmly attached to the vibrating platform and to each each of the stick using Epoxy cement. Measurements of the vibration amplitude were made while the stick was held in the outstretched hand and at the fingertip during stick balancing. These measurements are summarized in Table 1. The range of frequencies and amplitudes of the fingertip vibration are well within the range of responses recorded for human mechano–receptors [59]. We allowed the subjects to adjust their comfort level by self–selecting the degree of flexion at their knee (Figure 1).

**Virtual stick balancing measurements** involved using a paradigm developed previously that involves the interplay between a human and a computer [60], [61]. Briefly, the subject views a target and a dot on a computer screen. The dot reflects the movements controlled by the computer mouse and the movements of the target are controlled by the computer. The task is for the subjects to keep the dot and target as close together as possible while avoiding escape of either off the screen. The analogy to real stick balancing is made by programming the computer to move the target within a parabolic potential that is centered on the mouse position (see [60], [61] for more details). Computer programs were written in Python using VisionEgg, a high level interface between Python and OpenGL [62].

**Mechanical stick balancing measurements** involved using a paradigm that incorporates a dc–motor–operated plotter [15]. The pendulum is attached to a slider by means of a pivot: the pendulum can rotate freely in the 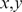
–plane and the cart is confined to move along the plotter rail in the 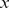
–direction. A potentiometer placed at the fulcrum of the pendulum detects 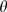. A dc servomotor drives the slider on the rail using a timing belt, and the position of the slider is detected by using a second potentiometer. Although it is possible to use separate proportional–integral–derivative (PID) controllers to stabilize 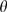 and the position of the slider, we controlled only 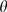 (see [15] for more details). The time delay was introduced by first digitizing the analog signal from the potentiometer and writing this information to a static random access memory (RAM). The contents of the RAM were read out after a time delay, 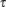, and converted to analogue to produced the output signal.

### Statistical and mathematical analyses

Since stick survival times are Wiebull–distributed we used non–parametric statistics, specifically a Mann-Whitney U test (Wilcoxon rank sum test), to test for statistical significance between stick survival curves. Cross–correlation functions, 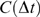 were converted to white noise by calculating the difference between consecutive time points [63] (‘diff’ function in MATLAB). The vertical displacement angle, 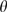, was calculated from the horizontal coordinates of the two ends of the balanced stick, i.e.
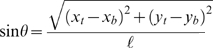
where the subscripts 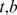 denote, respectively, the top and bottom of the stick. The change in fingertip speed, 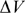, was calculated as follows [30], [31]: The change in the position of the marker, 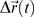, in one time step, 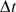 is
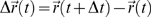
where the notation 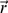 denotes the position vector measured from a common reference supplied by the Qualisys measurement system. The magnitude of the mean speed, 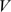, is
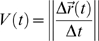
where the notation 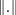 denotes the norm. Hence
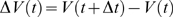
where 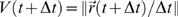. All computer simulations were performed using XPPAUT [64].
